# Draft Genome Sequence of an NDM-1- and KPC-2-Coproducing Hypervirulent Carbapenem-Resistant Klebsiella pneumoniae Strain Isolated from Burn Wound Infections

**DOI:** 10.1128/genomeA.00192-18

**Published:** 2018-03-29

**Authors:** Dan-Dan Wei, La-Gen Wan, Yang Liu

**Affiliations:** a Department of Bacteriology, First Affiliated Hospital of Nanchang University, Nanchang University, Nanchang, People’s Republic of China

## Abstract

We report here the draft genome sequence of an NDM-1- and KPC-2-coproducing hypervirulent carbapenem-resistant Klebsiella pneumoniae strain, isolated from a 58-year-old male in the People’s Republic of China with a burn injury.

## GENOME ANNOUNCEMENT

The hypervirulent Klebsiella pneumoniae (hvKP) bacterium, characterized by the ability to produce the hypermucoviscous phenotype, is known to cause life-threatening infections, such as pyogenic liver abscess, pneumonia, and meningitis (1). They are associated with several virulence factors compared to those of the classical K. pneumoniae strains, which include *rmpA*, *rmpA2*, *magA*, siderophores, such as aerobactin, enterobactin, and yersiniabactin, and genes coding for allantoin metabolism (2). Except for an intrinsic resistance to ampicillin, most hvKP strains are susceptible to commonly used drugs (3). Due to the dissemination of mobile genetic elements encoding the KPC, NDM, and OXA-48 types of carbapenemase (4), however, antibiotic-resistant hvKP has begun to emerge in the past few years (5), posing a threat to human health.

Here, we report the draft genome sequence of K. pneumoniae strain NUHL30457, recovered from wound secretions from a 58-year-old male burn patient hospitalized at the teaching hospital of Nanchang University, People’s Republic of China, in 2016. The strain belongs to the K2 serotype with a co-occurrence of NDM-1 and KPC-2 genes and is resistant to multiple clinically used antibiotics, including all β-lactams (ertapenem, meropenem, and imipenem had an MIC of 32 µl/ml), fluoroquinolones, aminoglycosides, sulfonamides, and macrolides.

The genomic DNA from K. pneumoniae NUHL30457 was sequenced by next-generation sequencing using an Illumina HiSeq 2000 instrument with a 2 × 150-bp paired-end approach. The draft genome of K. pneumoniae NUHL30457 comprises 5,302,595 bp with a GC content of approximately 58.45%. The protein-coding regions were predicted by Glimmer version 3.02 (http://www.cbcb.umd.edu/software/glimmer). In total, 5,496 coding genes were identified for a total length of 4,957,677 bp and an 85.73% coverage of the genome. Eighty-six tRNA genes and 25 rRNA genes had putative functions assigned on the basis of the annotation.

The contigs were initially annotated using Rapid Annotation using Subsystem Technology (RAST) (http://rast.nmpdr.org). A BLAST analysis and manual annotation utilized previously reannotated reference sequences and IS Finder (https://www-is.biotoul.fr). The multilocus sequence typing (MLST) and ResFinder (http://www.genomicepidemiology.org) databases were used to find the sequence type and antibiotic resistance genes present in the isolate. Virulence genes were defined with the help of the BIGSdb database (http://bigsdb.pasteur.fr/perl/bigsdb/bigsdb.pl?db=pubmlst_klebsiella_seqdef_public&page=downloadAlleles).

The capsule is an important virulence factor of encapsulated pathogens, including K. pneumoniae (6). Notably, many reports have shown that K1 and K2 serotypes are strongly associated with hvKP (1, 7). The strain was confirmed as serotype K2 belonging to sequence type 86 (ST86). Since the isolate was multidrug resistant, it was found to carry several genes coding for antimicrobial resistance against aminoglycosides, quinolones, and β-lactams. The genes *acc(6’)-Ib-cr* and *qnrS1*, coding for both fluoroquinolones and aminoglycosides, were also found. Surprisingly, among the genes encoding carbapenem resistance, the *bla*_NDM-1_ and *bla*_KPC-2_ genes are harbored in K. pneumoniae NUHL30457. Furthermore, the virulence-encoding genes *rmpA* (regulator of the mucoid phenotype A gene), *rmpA2*, *iroN*, *aerobactin*, and *mrkD* were detected in K. pneumoniae NUHL30457, which may have contributed to the infection and/or colonization of K. pneumoniae NUHL30457.

### Accession number(s).

The whole-genome shotgun project of K. pneumoniae NUHL30457 has been deposited at GenBank under the accession number CP026586.
